# A Case of Traumatic Tetanus Successfully Treated

**Published:** 1841-02

**Authors:** E. F. Wilson

**Affiliations:** Louisville, Ky.


Art. III.—*A Case of Traumatic Tetanus successfully Treated.* By E. F. Wilson, M. D., of Louisville, Ky.

On the 6th of July last, about 10 o'clock, P. M., I was called in great haste to see a female named Lavinia Hoover, aged about 19, robust, and of full plethoric habit. She was reported to me as being in a fit. I supposed it to be hysterical, but immediately obeyed the summons.

On entering the room where she lay I was at once by her appearance and attitude satisfied of my error, in supposing it hysteria, and the distortion of the countenance, the wrinkled nostrils and her position, which was bent backward, induced me to suspect she was suffering under a tetanic affection. This, I was informed, was her twelfth paroxysm. On making some examination I found the iris insensible to the light; the abdominal muscles very tense, and those of the back strongly contracted. Her breathing was rather slow, the pulse nearly natural, if any thing, slightly excited.

Appearances induced me to question her attendant closely, as to whether she knew of any injury received by the patient within a short time previously; and my suspicions were fully confirmed by the following account given to me:

Between three and four months previously, while walking barefooted, she had trodden on a part of a broken phial, and wounded the left foot, about half an inch behind the ball of the foot. The injury had been dressed at the time by a medical gentleman, who also examined it but did not discover any fragments of the glass, the patient being unwilling to allow a thorough examination. It healed pretty well, but the part always continued tender, so that in walking the patient could not put the foot flat to the ground. Her general health was very good, and she still continued moving freely about, though occasionally complaining of some uneasiness in the part. At last, about the 3d of July, she felt an unpleasant sensation in the neck extending upward, and a slight degree of stiffness in the motion of the jaw. On the 6th, she had been using much exercise in walking about, and had also been exposed to damp in washing or some household work, when in the afternoon she complained of considerable increase in the pain of the foot, and of stiffness in the neck and jaw. She was then suddenly seized with a violent spasm, or, as those around her supposed, a fit, was carried up to bed, and spasm after spasm succeeded until I was sent for.

On examination of the foot I found the wound almost entirely closed, though with still some appearances of inflammation about it. I had not at hand any instruments with which to fulfil the pressing indication; namely, the dilatation of the wound, and the searching for and removal of any fragments of glass which I believed must be deeply imbedded there; nor had I immediately at command any of the remedies commonly relied on, in such cases, to fulfil the second indication, namely, to relax the violent and involuntary contractions of the muscles. I determined, however, in the language of Mr. Gooch, "to stand to my guns." Something must be done immediately, or the patient must probably die. I fortunately recollected that I had with me some tartar emetic.

Of this I administered about grs. xv. in two doses, not with a view of producing emesis, for in the existing condition of things I did not believe this could be effected, nor was it. I now awaited its effects. In the course of an hour the muscular contractions had almost subsided, and great prostration ensued; the extremities becoming entirely cold. For this I had prepared, meanwhile, by having mustard at hand. I now applied sinapisms to the ankles, wrists, and epigastrium, and in a short time had the satisfaction of finding reaction had commenced. No unpleasant symptoms following, I directed the following prescription to be procured and given at once:

℞. hydrarg. submur. ℈i.

pulv. opii grs. ii., and an emollient poultice to be applied to the foot, and renewed, and left the patient for the night.

*July 7th.* Found her much better—had no return of the spasms and rested tolerably well during the night; she had had two dejections from the action of the medicine; her skin was tolerably moist; pulse regular; tongue slightly furred. The poultice, however, had not been applied as I ordered. Notwithstanding this omission, I now cut down and dilated the wound, and extracted four fragments of broken glass of more than a quarter of an inch in size; again ordered a poultice, together with some mild purgative medicine and left her.

8*th.* Patient still improving. No return at all of spasms; bowels open; pulse and skin pretty natural; had slept well. Ordered poultice to be continued with mild aperients. From this time she steadily improved, and on the 7th day was walking about, apparently in as good health as usual.

It appears to me, that I may attribute the prompt and complete success of the treatment to the antimony, for as soon as its action commenced, there was an immediate alleviation of all the urgent symptoms; the muscles became evidently much relaxed, and after the exhibition of the second dose, but a few minutes elapsed before there was a complete cessation of their contractions. The calomel and opium were not given until after reaction from the effects of the antimonial had taken place.

I have not met with an account of any case where a similar course has been pursued in this disease ; and altough it was so successful in the present instance, I would scarcely urge a like employment of this remedy except in the last resort. Yet the impressions left on my own mind, by its effects here, are such, that were I called to another case I should be tempted to use it in the same way, in preference to almost any other remedy.

The principal objection that could be urged against it, is the danger of exciting severe inflammation in the stomach. For such an emergency the practitioner might be prepared, by having at hand the means necessary to meet and combat it.

Several months have now elapsed, and excepting a slight attack of intermittent fever, the subject of the above remarks has enjoyed uninterrupted health.

*Louisville, Dec.* 20*th*, 1840.

